# Notes and News

**Published:** 1905-07-08

**Authors:** 


### NOTES AND NEWS.

The King and Queen, accompanied by Princess Victoria, inspected the new military hospital on Saturday, which has been erected at Millbank for the accommodation of invalids from the whole of the London garrison. The hospital is built in a series of blocks and stands by the side of the Tate Gallery. When completed it will have accommodation for about 230 patients. The royal visitors inspected the wards, the *x*-ray room, laboratory, and kitchens, and finally the site for the nurses' home.

From Uganda, Lake Victoria, comes a terrible account of the devastation created by sleeping sickness. As many as 87,000 victims are reported.

The Bristol Carnival in aid of the Bristol Royal Infirmary was held from June 26 to July 2. The resources of the Zoological Gardens were utilised to the utmost to secure an attractive setting to the fête, which we hope has achieved its object and added very considerably to the funds of the hospital.

The Walthamstow Urban District Council, at the instance of the Lea Conservancy Board, have been fined £50, with costs, for the pollution of the river Lea. The council have sewage works close to the river, and discharged some of the effluent into a brook running into the river. The Leyton Urban District Council were also summoned, but the charge was dismissed.

A very interesting ceremony in the form of a visit of general inspection of the wards and buildings of the St. Pancras Infirmary by the Lord Mayor, Aldermen, and a large body of visitors, took place on Friday, June 30th. Bouquets were presented to the Lady Mayoress and Mrs. Eves, wife of the Chairman of the Infirmary Committee. Mr. Eves said it was most desirable that the Council and the borough should work harmoniously together for the common good of St. Pancras. No greater compliment could be paid to their indefatigable Mayor, Mr. Purchese, than to say that owing to his and his three predecessors' untiring energy and devotion the rates of St. Pancras had known no increase.

The Hospital Sunday Fund collection now exceeds £35,000.

Another prosecution for the sale of unsound fruit has taken place. In this instance a hawker was sentenced to one month's imprisonment in default of a fine.

The annual inspection of the Gordon Boys' Home at Chobham took place on June 29th. The prizes were presented by Mrs. Moffatt, sister of General Gordon. There are now 250 boys in the Home.

A meeting of the council of the Convalescent Homes Association was held at 20 Hanover Square, W., on Thursday, July 6th, at 4 p.m. (Sir E. Hay Currie in the chair), to consider the draft rules and constitution which have been prepared by the executive committee and settled by counsel.

A bazaar in aid of the Homes for Working Boys was held at Sir George Newnes' residence on June 30 and July 1. Princess Louise of Schleswig-Holstein opened the bazaar on the first day, and the Earl of Aberdeen on the second day. The gardens were arranged as a Swiss village in mid-winter. There was a good display of carving and other woodwork executed by the boys, who now number in the home 340.

The Archbishop of Canterbury distributed prizes to the students of St. Thomas's Hospital Medical School on the 28th ult. The chairman alluded to the intention to open new wards shortly, and referred to the difficulty of providing funds for the maintenance of medical schools. The Archbishop pointed out that never before had so much been expected of the medical profession as in the present day, and he commended the readiness with which the discoveries in medical science were placed at the disposition of all.

The following is the text of regulations issued by the Postmaster-General in regard to the transit of pathological material through the post: "The transmission of pathological specimens is sanctioned only on the condition that they are handed in at a Post Office for transmission by registered letter post, and that they are packed in accordance with the regulations published in the Post Office Guide. These regulations, which are necessary for the protection of Post Office servants and of the public, provide that any deleterious liquid or substance sent by post must be enclosed in a receptacle hermetically sealed, which receptacle must itself be placed in a strong wooden, leathern, or metal case in such a way that it cannot shift about, and with a sufficient quantity of some absorbent material (such as saw-dust or cotton-wool) so packed about the receptacle as absolutely to prevent any possible leakage from the packet in the event of damage to the receptacle. The packet must be marked 'Fragile with care.' Any person who sends by post a deleterious liquid or substance for medical examination or analysis, otherwise than as provided by these regulations, is liable to prosecution, even if he is a patient sending something to his medical adviser for his opinion, or a medical practitioner sending something to a laboratory or elsewhere."

